# The effect of post‐extraction socket preservation laser treatment on bone density 4 months after extraction: Randomized controlled trial

**DOI:** 10.1111/cid.12991

**Published:** 2021-03-08

**Authors:** Aleksandra Križaj Dumić, Franja Pajk, Giovanni Olivi

**Affiliations:** ^1^ KRIŽAJ d.o.o Ljubljana Slovenia; ^2^ LA&HA ‐ Laser and Health Academy Ljubljana Slovenia; ^3^ Università Cattolica del Sacro Cuore di Roma Rome Italy; ^4^ InLaser Rome, Advanced Center for Esthetic and Laser Dentistry Rome Italy

**Keywords:** alveolar preservation, bone density, CBCT, laser post‐extraction procedure

## Abstract

**Background:**

Post‐extraction bone resorption may affect the outcome of ensuing restorations.

**Purpose:**

This study aimed to evaluate a comprehensive laser post‐extraction protocol by comparing resulting alveolar bone regeneration with that obtained after standard extraction procedure.

**Materials and Methods:**

About 53 simple extractions were randomized to either laser or control group. In the laser group, erbium (Er:YAG; 2940 nm) and neodymium (Nd:YAG; 1064 nm) lasers were used for degranulation, disinfection, de‐epithelialization of the surrounding gingiva, clot stabilization, and photobiomodulation. The primary outcome measure was change in bone density in the extraction area between day 1 and 4 months after extraction. Patients were monitored for potential side effects.

**Results:**

Increase in bone density at the follow‐up CBCT was significantly higher in laser than in control group (*p* < 0.001). No post‐operative pain, bleeding, or swelling was present in the laser group. In the control group, one patient had bleeding 3–5 days after extraction, two patients had swelling and three patients reported post‐operative pain rated 3–5 on a 0–10 pain scale up to 3 days after extraction.

**Conclusions:**

The proposed laser post‐extraction procedure is a safe and effective method to improve post‐extraction bone healing.

**What is known:**

- Recent systematic reviews suggested that laser photobiomodulation improves post‐extraction healing.
- Bactericidal and hemostatic effects of lasers may make them useful tools also in post extraction socket cleaning and disinfection, de‐epithelialization of the gingiva surrounding the extraction socket, and blood clot stabilization, but a comprehensive approach has not been studied.

**What this study adds:**

- This study is the first registered randomized controlled trial evaluating a comprehensive post‐extraction laser protocol by comparing resulting alveolar bone regeneration (measured from CBCT) with that obtained after standard extraction procedure.
- The laser post‐extraction procedure resulted in improved bone density and fewer side effects.

## 1 INTRODUCTION

Alveolar bone and soft tissue remodeling is a normal physiological response following tooth extraction.^1^ The resorption process varies amongst patients and tooth position and may be affected by several factors such as the presence of infection, previous periodontal disease, the extent of a traumatic injury, and the number or the thickness of the bony socket walls.^1^ An equilibrium is reached approximately 3–4 months post‐extraction.^1^ The clinical consequences of post‐extraction remodeling may affect the outcome of the ensuing therapies aimed at restoring the lost dentition, either by limiting the bone availability for ideal implant placement or by compromising the aesthetic result of the prosthetic restorations.^2^ Therefore, effective methods of reducing bone loss, accelerating bone healing, and making it more predictable are actively sought. Most studies focus on drugs or surgical techniques but more recently other modalities affecting the healing process have been investigated.^3^ Among them is the use of laser therapy.^3^

Photobiomodulation (PBM) is probably the best researched use of lasers in post extraction healing.^2^, ^4^ Recent reviews of accumulated animal and clinical studies reported that laser PBM therapy induced higher concentration of osteogenesis markers, as well as higher bone density and concluded that PBM improved the post‐extraction healing process, however, the results vary with laser wavelength and parameters used.^2^, ^4^ PBM with Nd:YAG laser has been found to improve healing after extraction in patients at high risk for osteonecrosis.^5^ Lasers also have other potential uses in the post‐extraction procedure. Use of Er:YAG laser for degranulation has been studied in periodontal and peri‐implant treatment and seemed to promote re‐osseointegration on contaminated implant surfaces to a greater degree than alternative methods.^6^ The advantages of laser degranulation are improved hemostasis and disinfection^6^ and Er:YAG laser may be safely used because its high absorption in water results in very efficient ablation with minimal thermal effect. This property of the Er:YAG laser also allows for very fine control of depth of ablation, which makes it highly suitable for fast and safe de‐epithelialization of the gingiva surrounding the extraction socket.^7^ This de‐epithelialization prevents ingrowth of epithelium into the socket and at the same time, produces an ablated rough surface, which may enhance retention of the blood clot.^6^

Blood clot is very important for proper uncomplicated socket healing.^8^ Laser irradiation of bleeding sockets may facilitate immediate clot formation and hemostasis.^6^ Different types of lasers and diodes have been used successfully to coagulate blood and prevent the loss of blood clot from extraction sockets in animal studies, resulting in improved alveolar bone preservation.^6^ In periodontal treatment, Nd:YAG laser has been demonstrated to be effective in fibrin clot stabilization in the periodontal pocket resulting in improved clinical outcomes.^6^ Bactericidal effect of laser therapy is considered advantageous for postoperative wound healing because lasers are capable of creating a disinfected field during surgery and reducing the risk of infection.^6^ In addition, because the Nd:YAG laser exhibits selective absorption in pigments, it is conceivable that this laser would be effective for devitalizing some of the pigmented bacteria, such as *Porphyromonas gingivalis*, that are associated with periodontal disease.^6^ This aspect may be particularly relevant for extractions performed due to periodontal disease. Moreover, lasers can ablate or inactivate toxic substances, such as bacterial endotoxins (lipopolysaccharide) which may positively influence wound healing of the treated site and offer several advantages over conventional mechanical treatment.^6^

Most clinical studies of the effect of laser therapy on post‐extraction healing have focused solely on photobiomodulation.^2^, ^4^ The aim of this study is to objectively evaluate a comprehensive post‐extraction laser protocol consisting of degranulation, disinfection, de‐epithelialization, clot stabilization, and photobiomodulation using Er:YAG and Nd:YAG wavelengths by comparing resulting alveolar bone regeneration (measured from CBCT) with that obtained after standard extraction procedure.

## 2 MATERIALS AND METHODS

### 2.1 Patients

Participants for this randomized clinical trial were recruited among patients attending our clinic between August and November 2019. Inclusion criteria were patients of either sex, aged 18–80 years, in whom simple tooth extraction was indicated, who agreed to participate in the study and signed informed consent. Exclusion criteria were pregnancy, use of photosensitizing medication, medication that would compromise bone healing, and complicated extraction. Extractions were randomized into laser and control groups (1:1) through drawing of closed envelops. The study was approved by the National Medical Ethics Committee (0120‐409/2019/5) and conducted according to the principles outlined in the Declaration of Helsinki. The protocol was registered at ClinicalTrials.gov (NCT04232202).

Power analysis was based on the primary outcome measure; the change in bone density. We assumed a large expected effect size of 0.9 for this study. We calculated that 21 teeth per group would be needed to have 80% power to detect such a difference with a test at two‐sided *α* = 0.05 (GPower 3.1 statistical software, Kiel University, Germany). Additional patients were recruited to account for uncertainties in effect size estimation and loss during follow up.

### 2.2 Procedure

Initial diagnosis was based on stomatological examination. Patient age, sex, number, location, and type of teeth extracted and indication for extraction were recorded. Local anesthetic (Scandonest 2%, 1.7 ml) was administered before extraction in both groups.

In the control group the standard post‐extraction procedure was carried out with cleaning of the post‐extraction socket with an alveolar spoon.

**FIGURE 1:**
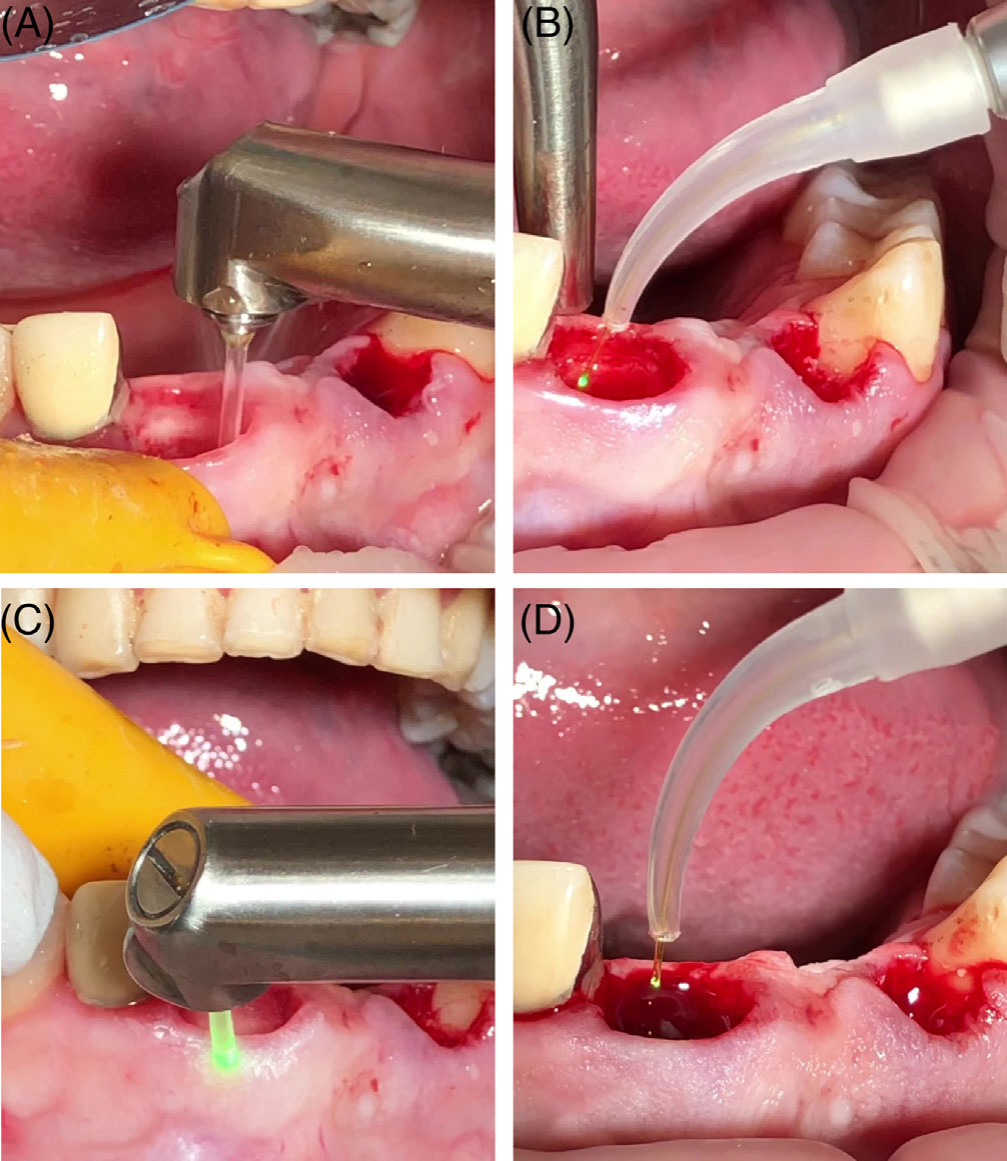
Laser post‐extraction procedure: (A) Er:YAG degranulation, (B) Nd:YAG disinfection, (C) Er:YAG de‐epithelialization, and (D) Nd:YAG clot stabilization

**FIGURE 2:**
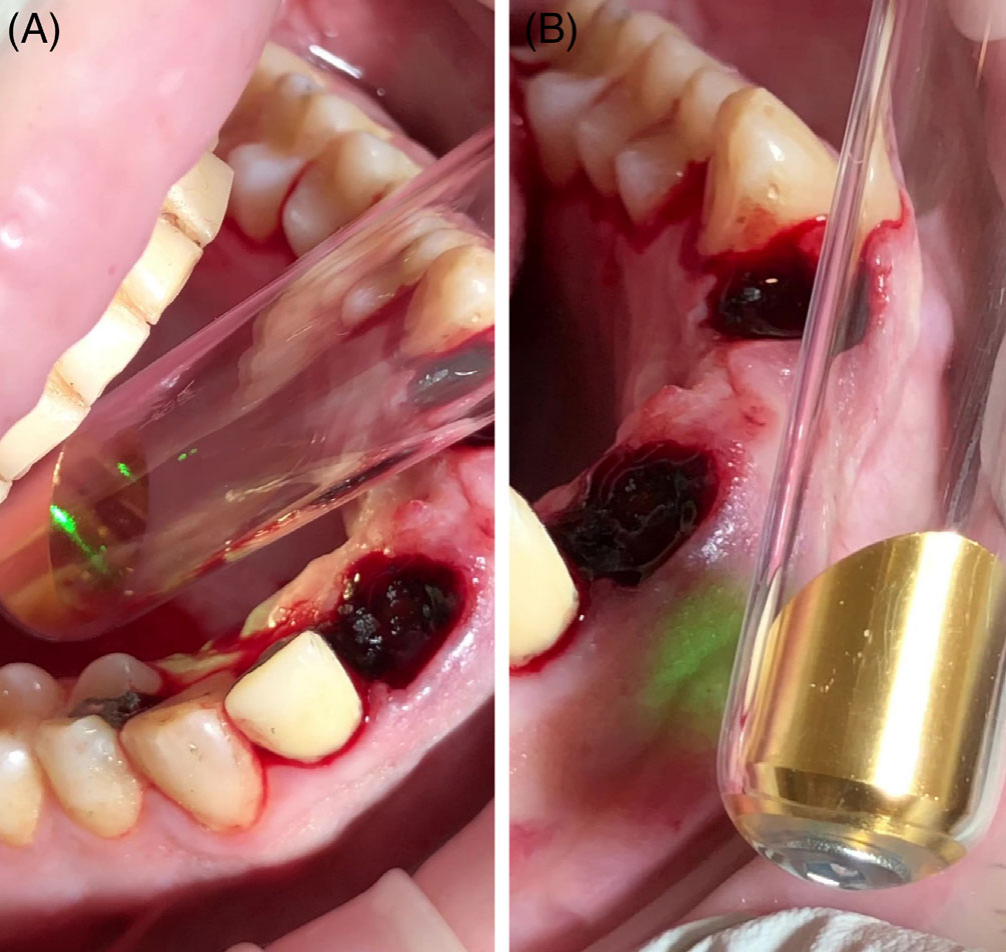
Nd:YAG photobiomodulation with intra‐oral angular adapter; oral (A) and vestibular (B)

In the laser group, Er:YAG and Nd:YAG lasers (LightWalker, Fotona, Slovenia; Figures 1 and 2) were used immediately after extraction for:

- *Degranulation*: Er:YAG, HC14 handpiece with cylindrical sapphire tip 1.3 mm diameter, SP pulse duration, 160 mJ, 15 Hz, Water/Air: 4/2 (Figure 1(A))
- *Disinfection*: Nd:YAG, 300 μm fiber, non‐contact, SP pulse duration, 2 W, 20 Hz (Figure 1(B))
- *De‐epithelialization* around 7 mm: Er:YAG, HC14 handpiece with cylindrical sapphire tip, 1.3 mm diameter, SP pulse duration, 120 mJ, 20 Hz, Water/Air: 4/2 (Figure 1(C))
- *Clot stabilization*: Nd:YAG, 300 μm fiber, non‐contact, VLP pulse duration, 4 W, 15 Hz (Figure 1(D))
- *Photobiomodulation*: Nd:YAG, Genova handpiece, 1 cm^2^ spot size, MSP pulse duration, 0.5 W, 10 Hz, 60 s oral (Figure 2(A)), and 60 s vestibular (Figure 2(B)), performed on the day of extraction and day 3, 5, and 7. All laser group patients included in the analysis received at least three of the four scheduled photobiomodulation sessions.

### 2.3 Outcome measures

- Blinded evaluation of bone volume and density from CBCT (Orthophos XG 3D, Sirona Dental Systems GmbH, Germany) images (at resolution 624x400 16bit grayscale, field 5 × 5.5 cm, 5.1 s exposure, 10 mA energy, dose: 159 mGy cm^2^) taken one day after extraction and after 4 months. The change in bone density in the area of extraction was the primary outcome measure. Analysis of CBCT images was performed with Galaxis (Sirona) and Somatom X.cite (Siemens Healthineers, Germany) program. Gray scale value (GSV) units were used.
- Monitoring for potential side effects (bleeding, pain, swelling, trismus).
- Pain during and after treatment on a scale of 0–10 (0‐no pain, 10‐intorellable pain).

### 2.4 Statistical analysis

Linear mixed model (as implemented in the function *lme* from the package *nlme* ^9^ in R statistical software^10^) was used to test the difference between laser and control group with change in bone density as the independent variable, intervention as the fixed factor and patient as the random factor, to account for non‐independence of teeth from the same patient. The influence of the following covariates on the results was tested in sensitivity analyses: patient age, sex, indication for extraction (periodontitis/granuloma/other), extractions adjacent to the extracted tooth (no/yes), extraction site (maxilla/mandible), tooth location (anterior/posterior), time after extraction (days), and initial bone density (GSV).

## 3 RESULTS

**FIGURE 3:**
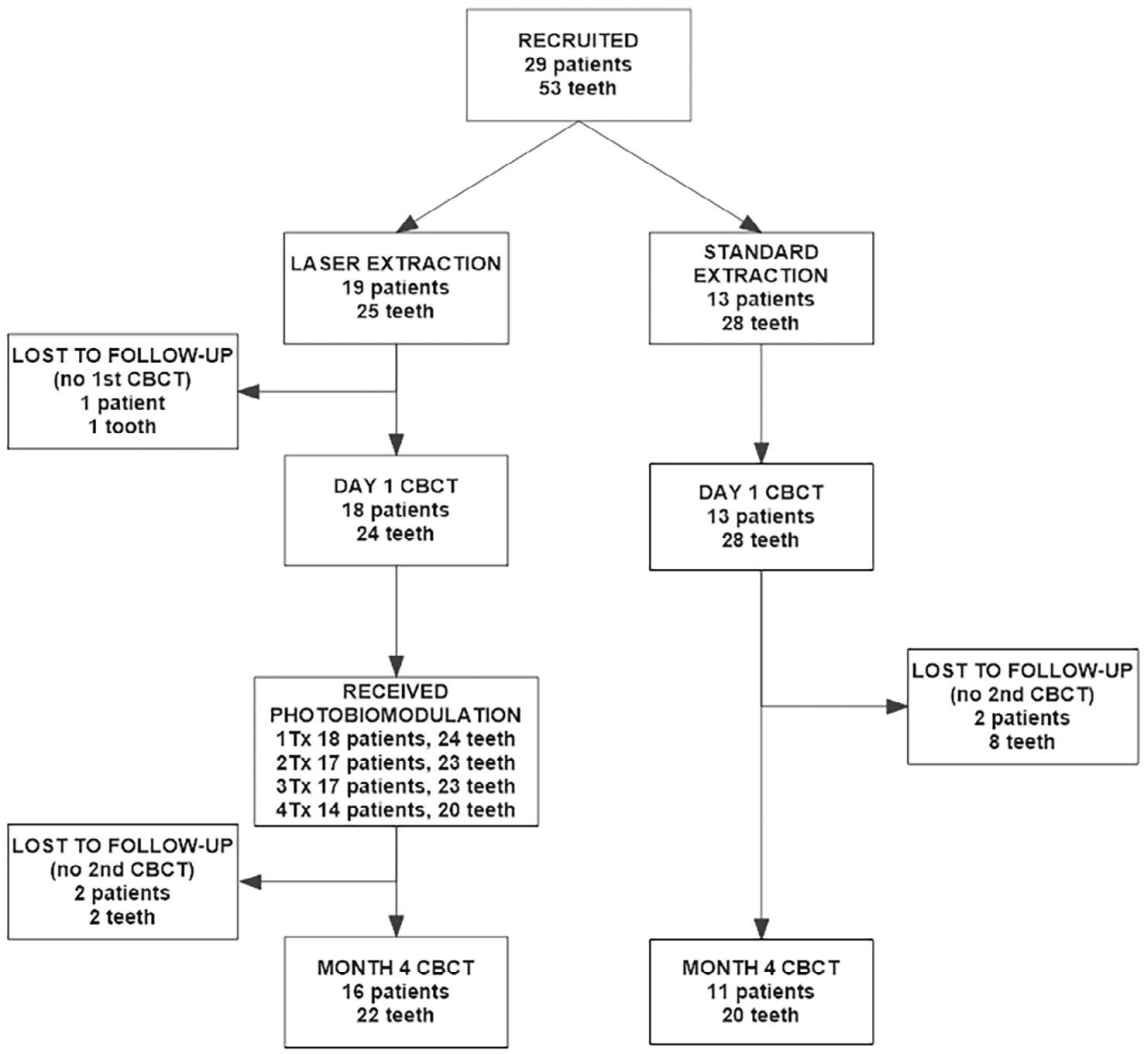
Trial flow‐chart. Three patients with multiple extractions had teeth in both groups

About 24 patients with 42 extracted teeth finished the study including the 4‐month CBCT scan (Figure 3). Patient demographics are presented in Table 1. The laser procedure takes about 5 minutes longer than the standard extraction procedure. No post‐operative pain, bleeding, or swelling was present in the laser group. In the control group, one patient had bleeding 3–5 days after extraction, which had resolved by day 7. Two patients in the control group had swelling for 3 days after extraction. Three patients in the control group reported post‐operative pain rated 3–5 on a 0–10 pain scale up to 3 days after extraction. No trismus was present in either group. No other side effects were observed or reported.

**TABLE 1:** Patient characteristics

Group	LASER	CONTROL
	16 patients	11 patients
	22 extractions	20 extractions
Sex (n [%])		
Male	5 (31%)	4 (36%)
Female	11 (69%)	7 (64%)
Age (mean ± SD)	56 ± 16	62 ± 14
Reason for extraction (n [%])		
Chronic periodontitis	13 (59%)	16 (80%)
Periapical granuloma	4 (18%)	2 (10%)
Vertical root fracture	3 (14%)	–
Horizontal root fracture	1 (5%)	–
Radix relicta	1 (5%)	–
Caries profunda	–	2 (10%)
Number of extractions per patient (n [%])		
1	12 (75%)	7 (64%)
2	2 (13%)	1 (9%)
3	1 (6%)	1 (9%)
≥4	1 (6%)	2 (18%)
Extraction site (n [%])		
Maxilla	18 (82%)	11 (55%)
Mandible	4 (8%)	9 (45%)
Tooth extracted (n [%])		
Incisor	6 (27%)	4 (20%)
Canine	1 (5%)	2 (10%)
Premolar	5 (23%)	5 (25%)
Molar	10 (45%)	9 (45%)

**FIGURE 4:**
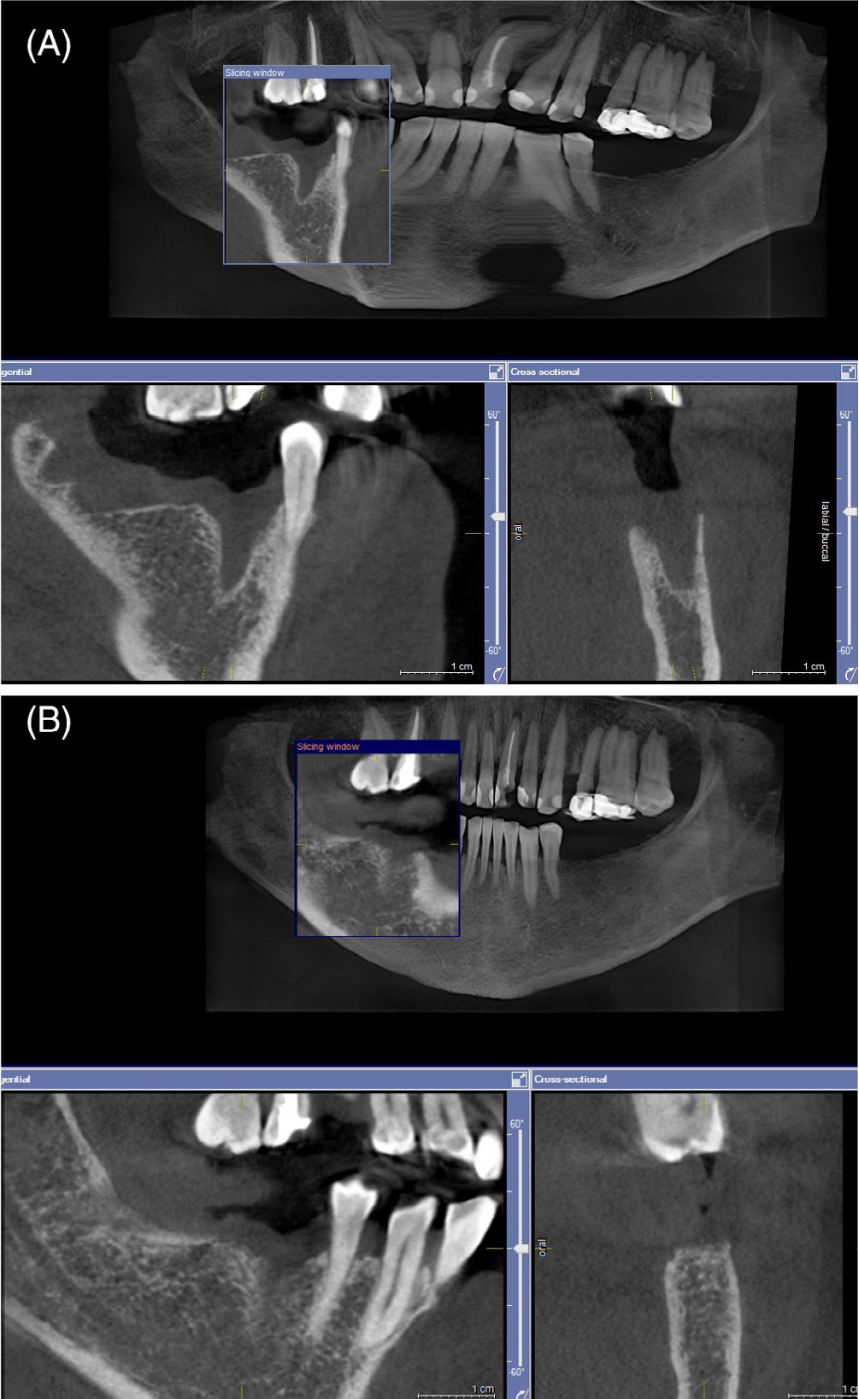
Female, 56 years old, extraction of 45 and 47 due to chronic periodontitis. Laser post‐extraction procedure was used. CBCT scan 1 day after extraction (A) and at for month follow up (B)

**FIGURE 5:**
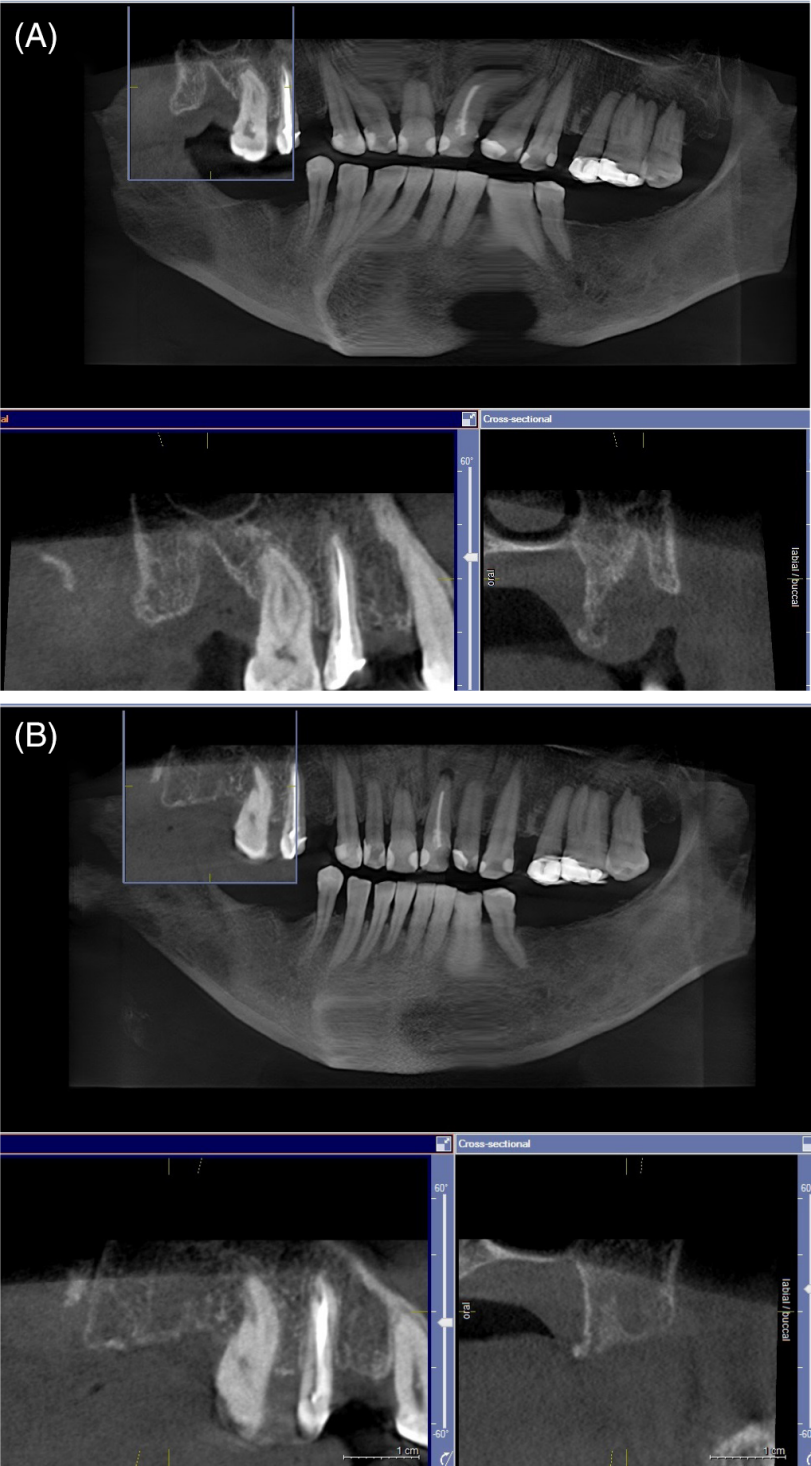
Same patient as Figure 4, extraction of 18 due to chronic periodontitis. Standard extraction procedure was used. CBCT scan 1 day after extraction (A) and at for month follow up (B)

**FIGURE 6:**
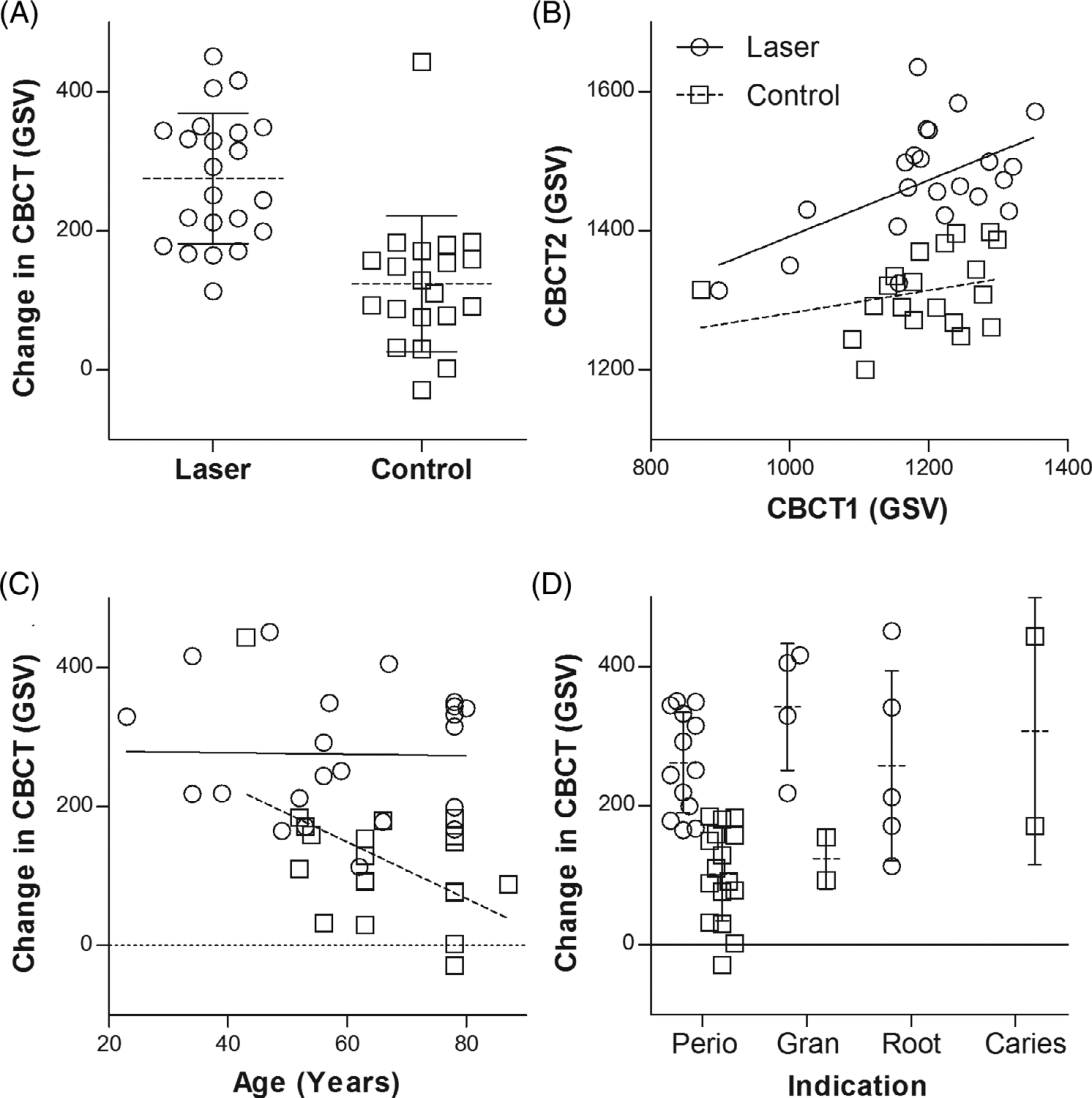
A, Change in gray scale values (GSV) between CBCT 1 day after extraction and at 4 months follow‐up. Individual observations are represented by symbols. The dotted line with error bar represents mean and standard deviation. B, Relationship between GSV of initial (CBCT1) and follow‐up CBCT scan (CBCT2). C, Relationship between change in GSV and patient age. While bone healing appears to decrease with age in the control group (dotted line) it remains constant in the laser group (solid line). D, Change in GSV according to indication for extraction; chronical periodontitis (Perio), apical granuloma (Gran), vertical or horizontal root fracture or radix relicta (Root), or caries profunda (Caries). Symbols as in (A)

The follow‐up CBCT was taken between 3 and 5 months after extraction (mean 4.2 ± 0.4 months). Better bone healing was noted with the laser procedure (Figure 4) than with the standard post extraction procedure (Figure 5), as evidenced by higher increase in bone density (GSV) at the follow‐up CBCT in laser patients compared with control patients (Figure 6(A)). The difference was statistically significant (Table 2).

**TABLE 2:** Results of linear mixed model analysis of change in GSV values between CBCT 1 day after extraction (CBCT1) and at 4 months follow‐up

Predictor	p*	Coefficient	Estimate (95% CI)	
Intervention	<.001	Laser	147	(106 to 187)
Covariates:				
CBCT1 (GSV)	<.001		−0.6	(−0.8 to −0.4)
Age	.0851	>60	−45	(−94 to 4)
Sex	.1748	Male	−36	(−86 to 13)
Indication	.3471	Periodontitis	−16	(−76 to 44)
		Other	34	(−37 to 104)
Adjacent extractions	.1872	Yes	41	(−10 to 93)
Arch	.4035	Maxilla	−19	(−68 to 30)
Location	.9411	Posterior	−2	(−44 to 40)
Interval (Days)	.8396		−0.4	(−2 to 2)

*Notes:* Extractions were the unit of analysis and patient was the random factor. *p value was obtained by likelihood ratio test comparing the full model with the full model without the specified predictor.

The effect of laser treatment was statistically significant regardless of inclusion or exclusion of covariates. Results of the full model with all covariates included are presented in Table 2 and show a significant effect of laser treatment even when all potentially relevant factors are controlled for. The only statistically significant covariate was GSV value at initial CBCT. Figure 6(B) shows that final bone density was higher in the laser group regardless of the initial bone density.

Coefficient estimates also indicate slower bone healing in patients 60 years old or older but the confidence interval is too wide to draw conclusions (Table 2). The effect of patient age on change in bone density differed between groups (Figure 6(C)). Bone healing decreased with age in the control group but not in the laser group, indicating that older patients may benefit even more from laser treatment.

It was not possible to explore the effect of the indication for extraction on the outcome, because most patients in both groups had teeth extracted due to periodontal disease (Table 1, Figure 6(D)). The positive effect of laser on bone healing was statistically significant in this subgroup (Effect: +155 ± 22 GSV, 95% Confidence interval: 107–204 GSV, *p* < .001).

## 4 DISCUSSION

The results of this study show that a comprehensive laser post‐extraction procedure consisting of degranulation, disinfection, de‐epithelialization, clot stabilization and photobiomodulation (PBM) using Er:YAG and Nd:YAG lasers significantly improves bone healing at 4 months post‐extraction. There were no side effects in the laser group in contrast with the control group where post‐operative bleeding, swelling, and/or pain were recorded in 9 out of 11 patients.

Most clinical studies of the effect of laser therapy on post‐extraction healing have focused solely on PBM.^2^, ^4^ Previous clinical PBM studies of post‐extraction wound healing provided mixed results, possibly due to a wide variety of wavelengths and protocols used.^4^ Another shortcoming of these studies may be inappropriate intervals or periods of observation, with follow up that was either too early (7–40 days) or too late (6 months) to detect the potential accelerating effect of PBM on post‐extraction bone healing.^2^ The results obtained with Nd:YAG PBM are more consistently positive, possibly due to deep penetration depth of this wavelength. Nd:YAG laser irradiation after tooth extraction promotes osteoblast differentiation, as demonstrated by the higher expression of osteocalcin in experiments in rats.^11^ Observational studies of complications after tooth extraction in patients either at risk of medication related osteonecrosis of the jaw (MRONJ)^12^ or patients with history of MRONJ^5^ showed that Nd:YAG PBM reduces the risk of complications. Studies comparing the outcome of guided tissue regeneration alone or in combination with Nd:YAG PBM for treatment of furcation defects or periodontal defects showed significantly more improvement in pocked depth, clinical attachment level, horizontal probing depth, and alkaline phosphatase levels in lased than in non‐lased group.^13^, ^14^

In addition to PBM, the laser post‐extraction protocol used in this study consisted also of Er:YAG debridement, Nd:YAG disinfection, Er:YAG de‐epithelialization, and Nd:YAG clot stabilization. These steps have proven beneficial in peri‐implant and periodontal therapy, improving the healing of periodontal pockets and bone defects,^6^ but are rarely used in post‐extraction studies.^15^ Further studies would be needed to determine the contribution of each step to the final result. Use of barrier membranes has significant positive effects on the outcomes of alveolar ridge preservation,^16^ indicating that clot stabilization and prevention of epithelial ingrowth are important contributing factors in the final result. Examples from periodontitis research suggest that a combination of Nd:YAG and Er:YAG lasers is superior to Er:YAG laser alone.^17^

Four PBM sessions were scheduled for patients in the laser group in this study. Requirement for additional visits might produce problems with scheduling and patient compliance. Multiple sessions of Nd:YAG PBM are commonly performed, but a single Nd:YAG PBM session was shown to effectively reduce swelling and improve oral health related quality of life (OHRQoL) after sinus lift surgery compared with controls,^18^ and improve trismus and OHRQoL in acute pericoronitis.^19^ However, these are relatively short‐term effects compared with bone healing. A recent study comparing the effect of single versus multiple low‐level laser applications on bone formation in extraction socket healing in rabbits showed that the percentage of newly formed bone was higher in both laser groups compared with control extractions and that the difference between single or multiple (5 treatments over 12 days) laser application was not statistically significant.^2^

A shortcoming of this study is a relatively small patient group that was heterogeneous with respect to patient and tooth position related variables that may influence the healing process. Nevertheless, the result was robust regardless of the inclusion or exclusion of all possible covariates in the statistical model, providing confidence in our conclusions. One of the factors that may affect bone healing is patient age and best healing is observed in younger patients.^20^ As the laser group patients were somewhat younger on average this may have influenced the results. However, bone density 4 months after extraction decreased with increasing age in the control group, but not in the laser‐treated patients. Our findings suggest that older patients benefit more from laser treatment than younger patients.

Laser treatment may have been especially advantageous for the patients in this study, because most of them had teeth extracted due to indications related to a heavy bacterial load (chronic periodontitis or periapical granuloma). Chronic periodontitis as the reason for extraction was found to result in the worst bone healing while orthodontic indications result in the best bone healing after extraction without intervention.^20^ The benefits of laser treatment to reduce the bacterial load in chronic periodontitis are well researched.^6^ A recent randomized controlled trial showed a significant benefit of combined Er:YAG and Nd:YAG laser treatment over conventional scaling and root planning in treatment of periodontitis.^17^ Nd:YAG laser was used for pocket disinfection followed by root debridement with Er:YAG laser and subsequent application of Nd:YAG laser for blood clot stabilization.^17^ The combined laser treatment resulted in the highest reduction of all bacteria count after six months (93.0%), followed closely by Er:YAG laser alone (84.9%), whereas SRP (46.2%) failed to reduce *Treponema denticola*, *Peptostreptococcus micros*, and *Capnocytophaga gingivalis*. Full mouth plaque score and bleeding on probing scores dropped after 6 months and were the lowest in both laser groups. The combination treatment resulted in higher periodontal pocket depth reduction and clinical attachment gain compared with SRP or Er:YAG laser alone in 4–6 mm deep pockets.^17^

An advantage of this study is use of an objective outcome measure. Bone density was estimated from voxel values (GSV) of CBCT images. There is some lingering controversy regarding the use of GSV for bone density evaluation since Hounsfield units (HU) are not directly applicable for CBCT.^21^, ^22^ Multiple studies have shown that when the same CBCT scanner is used, the grey value of scanned bone can be directly converted to the corresponding bone mineral density value using a linear calibration curve.^21^ High correlation has been found between voxel value of CBCT and CT.^21^ However, GSVs may shift owing to the use of different CBCT devices, exposure parameters, the position of the measurement in the field of view (centrally vs. peripherally), and the amount of mass inside and outside the field of view, introducing errors to quantitative estimates of actual bone density.^22^ Nevertheless, there is agreement that GSVs can be used to evaluate bone density in a relative way^21^, ^22^; by pre‐treatment and post‐treatment comparison of CBCT GSVs when scanning patients under the same exposure conditions, especially if changes in bone density are relatively large.^22^ Furthermore, bone quality evaluated by CBCT shows a high correlation with the primary stability of dental implants.^21^ CBCT grey scale evaluation of bone density is preferred over CT despite somewhat lower accuracy due to reduced cost and radiation dose.^21^

## 5 CONCLUSION

A comprehensive laser post‐extraction protocol using Er:YAG and Nd:YAG laser wavelengths for degranulation, disinfection, de‐epithelialization, clot stabilization, and photobiomodulation of the extraction socket proved to be effective in increasing bone density 4 months after extraction in comparison with controls, resulting also in fewer side effects. Our data suggests that addition of lasers to post‐extraction protocol may be especially beneficial for older patients and patients with chronic periodontitis. Reduced rate of complications and improved bone healing allow earlier restoration, support better aesthetic outcomes after prosthetic restorations and facilitate optimal placement of implants.

## CONFLICTS OF INTEREST

Dr. Pajk has nothing to disclose. Dr. Križaj Dumić has nothing to disclose. Prof. Dr. Olivi has nothing to disclose.

## AUTHORS CONTRIBUTION

All authors contributed to the conception and design of the study, Aleksandra Križaj Dumić: Contributed to acquisition of data; Franja Pajk: Preformed statistical analysis, all authors contributed to analysis and interpretation of data, all authors contributed to drafting the article and revising it critically for important intellectual content. All authors approved the manuscript and this submission.

## Data Availability

The data are not publicly available due to privacy restrictions.
